# Network pharmacology and bioinformatics to identify molecular mechanisms and therapeutic targets of Ruyi Jinhuang Powder in the treatment of monkeypox

**DOI:** 10.1097/MD.0000000000033576

**Published:** 2023-04-28

**Authors:** Xi Zhang, Xinping Yu, Zhichao Yu, Chengcheng Fan, Yueming Li, Huan Li, Yingkai Shen, Zijin Sun, Shuo Zhang

**Affiliations:** a College of Traditional Chinese Medicine, Shandong University of Traditional Chinese Medicine, Jinan, China; b College of Traditional Chinese Medicine, Beijing University of Traditional Chinese Medicine, Beijing, China; c College of Rehabilitation, Shandong University of Traditional Chinese Medicine, Jinan, China; d College of First Clinical Medical, Shandong University of Traditional Chinese Medicine, Jinan, China

**Keywords:** bioinformatics, monkeypox, molecular mechanisms, network pharmacology, Ruyi Jinhuang Powder, therapeutic targets

## Abstract

Monkeypox outbreaks across the globe has aroused widespread concern. Ruyi Jinhuang Powder (RJP), a common formula in Chinese medicine, is used to treat pox-like illnesses. This study aimed to identify the molecular mechanisms and therapeutic targets of RJP for the treatment of monkeypox using network pharmacology and bioinformatics techniques. The bioactive substances and potential targets of each component of RJP were retrieved from the Traditional Chinese Medicine Systems Pharmacology Database and Analysis Platform (TCMSP). The differentially expressed genes (DEGs) of the monkeypox virus (MPXV) were identified from the GSE24125 by GEO2R. Key signaling pathways, bioactive components, and potential targets were obtained by bioinformatics analysis, including gene ontology (GO) and Kyoto Encyclopedia of Genes and Genomes (KEGG), disease ontology (DO), and protein-protein interactions (PPI) analyses. Finally, molecular docking was used to predict the interaction between active compounds and core targets. A total of 158 active ingredients and 17 drug-disease-shared targets of RJP were screened. Bioinformatics indicated that wogonin and quercetin might be potential drug candidates. Potential therapeutic targets were identified. Immune-related mechanisms that exerted antiviral effects included signaling pathways like TNF, age-rage, and c-type lectin receptor pathways. Our results illustrated the good therapeutic effect of RJP on monkeypox in terms of biological activity, potential targets, and molecular mechanism. This also offered a promising strategy to reveal the scientific basis and therapeutic mechanism of herbal formulas used to treat the disease.

## 1. Introduction

One of the 4 orthopoxviruses that are pathogenic to humans, along with the smallpox virus, is the monkeypox virus (MPXV). It is a double-stranded DNA virus belonging to the Orthopoxvirus genus of the family Poxviridae. The most important symptom of monkeypox is the rash. It may also result in clinical symptoms such as fever, chills, and muscle aches.^[1]^ Although the rash passes through multiple stages before it eventually scabs over and falls off, there is a strong likelihood that it may leave scars. The death rate from monkeypox can reach 10% when sepsis, meningitis, and osteomyelitis are also involved.^[2]^ According to reports, over 9109 confirmed cases of monkeypox have been found in at least 40 non-African nations in Europe, Latin America, and Asia, as of July 9, 2022 (https://www.monkeypoxmeter.com/).^[3]^ There is no specific treatment for monkeypox. It has been estimated that smallpox vaccination with vaccinia virus (another orthopoxvirus) protects against monkeypox by approximately 85%.^[4]^ However, in 1980 routine vaccination against smallpox became obsolete due to its eradication,^[5]^ which recently contributed to the geographic spread of monkeypox cases. Therefore, it is imperative to seek out treatment for monkeypox aggressively.

Since monkeypox is not a large-scale epidemic, there are no records of Chinese medicine treating monkeypox in the past and present. However, the Chinese have been treating pox diseases with traditional Chinese medicine (TCM) for thousands of years and have accumulated rich clinical experience. After careful screening, we proposed Ruyi Jinhuang Powder (RJP) as a potentially effective formula for treating monkeypox. RJP is derived from Chen Shigong “Surgical Authenticity” from the Ming Dynasty and is included in the 2015 edition of “Chinese Pharmacopoeia.” The ten different types of herbs that make up RJP are Baizhi (radix angelicae dahuricae), Dahuang (radix et rhizoma rhei), Houpu (cortex magnoliae officinalis), Huangbai (cortex phellodendri), Chenpi (percarpium citri reticulatae), Jianghuang (rhizoma curcumae longae), Cangzhu (rhizoma atractylodis), Tiannanxing (rhizoma arisaematis), Tianhuafen (radix trichosanthis kirlowii), and Gancao (radix glycyrrhizae). Huangbai and Cangzhu are the Jun herbs in the entire formula, which are also ingredients in the traditional Chinese medicinal formula Ermiao San. They have the function of clearing heat and drying dampness. The Chen herbs, which include Chenpi, Tiannanxing, Dahuang, and Houpu, clear heat and dry dampness, resolve phlegm and move qi. Jianghuang breaks blood and moves qi. Tianhuafen and Baizhi clear heat, detoxify toxins, reduce swelling, and relieve pain as adjuvants. Gancao harmonizes all the herbs, clears heat, and detoxifies while astringes the sores. Combining all the herbs can detoxify and subdue swelling, clear heat and astringent sores. RJP is frequently used to treat dermatological diseases, including sores, ulcers, and psoriasis, and has the effect of clearing heat, detoxifying, and dispersing swelling and nodules. We selected RJP for the following reasons: First, in ancient Chinese medical texts, monkeypox, like smallpox, is classified as an epidemic disease. The goal of the therapy should be to clear the heat, detoxify the toxin, resolve the dampness, and move the qi. This is precisely aligned with the formula meaning of RJP. Second, clinical studies reported that RJP could lower the serum levels of cytokines such as c-reactive protein, interleukin-6, and soluble interleukin-2 receptor as well as significantly improve the clinical symptoms of mumps, senile skin ulcers, and acute herpes zoster.^[6–8]^ Kai Hu noted that RJP could shorten the wound healing time of perianal abscesses and relieve the pain.^[9]^ Skin soft tissue infections of the can be treated more quickly and with less infectivity-related indices when RJP is used in combination with antibiotics.^[10]^ It can reduce the symptoms of lesions in people with papulopustular rosacea^[11]^ and has a positive clinical impact on various phlebitis.^[12–15]^ Therefore, RJP has accumulated a significant amount of clinical data for the treatment of several dermatophytosis diseases. Skin lesions are the most significant clinical sign of monkeypox and the one most likely to cause long-term effects. We concluded that RJP, following the theory of TCM discriminatory evidence and clinical research, is one of the preferred formulae for the treatment of monkeypox. However, its potential therapeutic mechanisms for treating monkeypox have not been fully understood.

Network pharmacology refers to a systematic pharmacological research method that combines pharmacology, molecular biology, electronic technology, and bioinformatics and holds great promise in studying the network relationships between the active ingredients in herbal formulas and associated targets, metabolic pathways, and diseases.^[16]^ This systematic approach to drug research is consistent with TCM holistic viewpoint and the multi-component, multi-pathway, multi-target synergistic mechanism of action of TCM formulations.^[17,18]^

**Figure 1.**
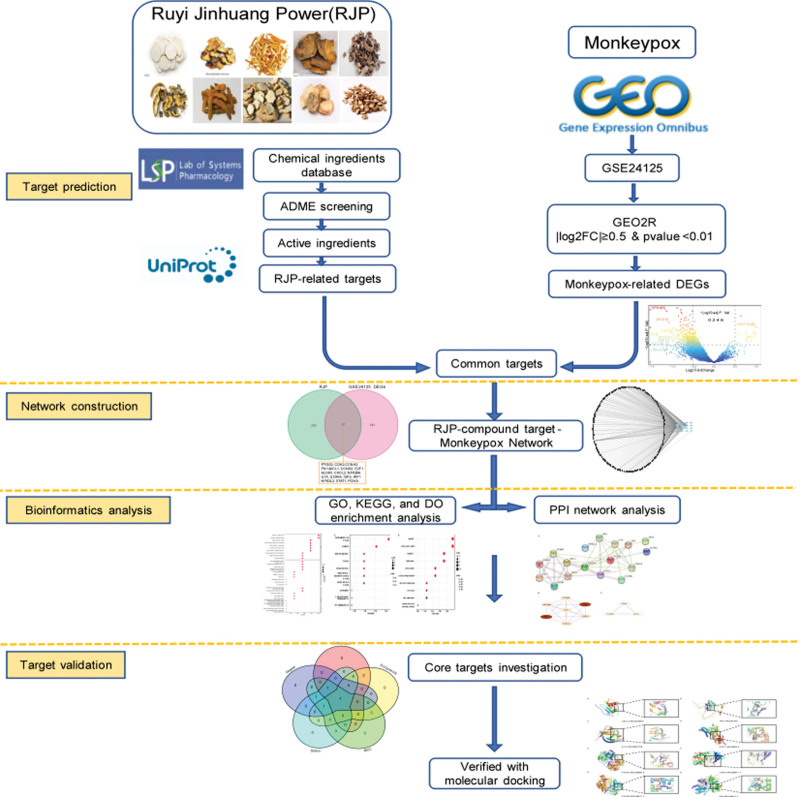
Technology roadmap for this study.

To determine the common target and potential mechanism of action of RJP in the treatment of MPXV and to develop new medications, this study combined network pharmacology, bioinformatics, and molecular docking technology to identify the bioactive components and targets of RJP. The technology roadmap for this study is presented in Figure 1.

## 2. Materials and methods

### 2.1. Collection of bioactive substances and prediction of related targets in RJP

The components of RJP and their corresponding targets were obtained from the Traditional Chinese Medicine Systems Pharmacology Database and Analysis Platform (TCMSP, https://old.tcmsp-e.com/tcmsp.php).^[19]^ Many medication ingredients cannot reach specific protein targets in cells due to the pharmacological properties of TCM. Therefore, selecting the appropriate screening index for network pharmacology was very important. The oral bioavailability (OB) value (systemic bioavailability of oral or absorption and distribution) is an essential pharmacokinetic parameter in the absorption, distribution, metabolism and excretion properties of drugs. The drug-like properties (DL) value (structural similarity of a compound to a clinically used drug in the DrugBank database, https://go.drugbank.com/drugs) is a significant screening value for pre-judging a large number of compounds in a short time. Therefore, only compounds that passed the screening requirements of OB ≥ 30% and DL ≥ 0.18 were included in the study. After filtering the active ingredients, it was important to match the target of each ingredient. To obtain the official gene name of the protein, we logged into the UniProt database (http://www.uniprot.org/),^[20,21]^ used the UniProtKB search function, and narrowed our search to “Homo sapiens.” Because this study does not involve the ethical issues of clinical and animal experiments, and it is a study about network pharmacology and bioinformatics, it does not need ethical approval.

### 2.2. Mining and prediction of monkeypox-related targets

The Gene Expression Omnibus (GEO) (https://www.ncbi.nlm.nih.gov/geo/) database^[22]^ is an open-source database that stores microarrays of various diseases of organisms. We downloaded the human genome data of monkeypox GSE24125 from the GEO database. GSE24125 is the gene set of a person infected with the MPXV contributed by Rubins.^[23]^ According to the original data provided by the contributors, we selected the primary human macrophage data on the GPL10912 platform (SMD Print_1046 LC-48 Homo sapiens), including 16 MPXV groups as the disease group, and the remainder of the inactivated virus group and the simulated infection group as the control group. The GEO2R online analysis tool^[22]^ performed ID conversion, miss value processing, and force normalization to remove the batch correspondence. The GEOquery and limma packages provided by GEO2R were used to read the data and obtain the difference multiple. According to the regulation, we set the threshold of differentially expressed genes (DEGs) as | log2FoldChange | ≥ 0.5 and the *P* value <.01. The DEGs that satisfied the above conditions were used for further analysis. The ggplot2 package was used to draw the gradient volcano map to depict the distribution and expression of DEGs.

### 2.3. Analysis of the target of RJP and MPXV

The collected targets of RJP were intersected with the targets of the MPXV, and the intersection targets were used as the common targets of RJP on the MPXV. The Venn diagram was drawn using the Venn package (version R 4.0.2). Drug composition target maps were obtained by Cytoscape 3.9.0 software.

### 2.4. Pathway enrichment analysis and disease association analysis

After converting the collected common targets into related Entrez IDs, we carried out Gene Ontology (GO), Kyoto Encyclopedia of Genes and Genomes (KEGG) pathway, and Disease Ontology (DO) enrichment analyses. We used R 4.1.0 and related R packages (clusterProfiler,^[24]^ org. Hs. e.g., db, enrichplot, ggplot2, pathview, DOSE,^[25]^ ggnewscale, stringr). Results with *P* values < .05 were normally regarded as significant. The 3 enrichment analysis methods displayed only the first ten results.

### 2.5. Protein-protein interaction data analysis (PPI)

We submitted the common action targets derived from the intersection of RJP and MPXV to the STRING database (https://cn.string-db.org/),^[26]^ which has the most organisms and proteins and set the operating conditions as Homo sapiens, to determine the core therapeutic targets. The confidence level was set to 0.4, and the independent nodes were hidden. The relevant PPI data was then downloaded from the STRING database and loaded into Cytoscape 3.9.0, extensively used in the construction and visualization of topological networks, particularly in network pharmacology research.^[27]^ The MCODE plug-in in Cytoscape software was used to analyze the core network and identify its closely linked sub-modules. The key modules were selected for further analysis, and the Degree, BottleNeck, EcCentricity, Maximal Clique Centrality, and Stress in the Cytohubba plug-in were used for further screening. The intersection of the top ten targets of each component was then used to determine the core therapeutic targets.^[28]^

### 2.6. Molecular docking

Molecular docking is commonly used to explore novel drugs, which can accurately predict the appropriate target binding site and small molecule ligand conformation and evaluate the binding affinity.^[29]^ In this study, candidate target proteins from the human species were screened for molecular docking. AutoDock tools 1.5.6 was used to process molecular docking in combination with AutoDock Vina, a widely adopted innovative molecular docking method that has been demonstrated to enhance the efficiency and accuracy of molecular docking. The docking was performed as follows:

In the first step, macromolecular receptor files were prepared from the Protein Data Bank. Protein data bank (http://www.rcsb.org/)^[30]^ was used to obtain 3D structures of proteins (if not available, UniProt was used). These structures were then imported into PYMOL software (https://pymol.org/2/) and modified by removing water molecules, co-crystal ligands, and ions. Additionally, AutoDock Tools 1.5.6 was used to add missing hydrogen bonds and kolman partial charges to incorporate the non-polar hydrogens into their carbon counterparts. They were stored as PDBQT protein receptor files. In the second step, the 2D structure of the compound was downloaded from the PubChem database (https://pubchem.ncbi.nlm.nih.gov/)^[31]^ and then loaded into Chem3D software to minimize and transfer the energy (MM2 force field) into the 3D structure. Subsequently, the resultant 3D structure was modified in the AutoDock tool 1.5.6 by adding hydrogen and protonation, and the non-polar hydrogen was rotationally merged with its corresponding carbon. The torsion was automatically set in the software. The structure was then saved as a PDBQT ligand file. In the third step, the PDBQT structure of the receptor and ligand were imported into the AutoDock tool 1.5.6 to construct the docking pairing pocket. When building the box, the center point was located on the macromolecule, and the number of points in the X, Y, and Z dimensions were set so that the box completely wrapped the protein. These parameters of the paired cassette were determined for each protein by the configuration file for the next interfacing with Vina, including box center (center X, center Y, center Z), box size (size _ X, size _ Y, size _ Z), massive docking (num mode) to output (set to 20), energy _ range (set to 5). Finally, Vina software was used for molecular docking to calculate the docking affinity. In this process, besides the configuration files, PDBQT files of proteins and ligands were also used because PDBQT files determined the information of docked ligands and proteins. After Vina operated, the results were exported as pdbqt * and log *.txt files. The binding affinity score of the target protein receptor to the small molecule ligand was established by scoring the free energy of docking.

## 3. Results

### 3.1. Collection of bioactive substances in RJP

We used OB ≥ 30% and DL ≥ 0.18 as the screening threshold to obtain the screened drug components, which included 20 components of Baizhi (radix angelicae dahuricae), 4 components of Cangzhu (rhizoma atractylodis), 6 components of Tiannanxing (rhizoma arisaematis), 5 components of Chenpi (percarpium citri reticulatae), 7 components of Dahuang (radix et rhizoma rhei), 88 components of Gancao (radix glycyrrhizae), 2 components of Houpu (cortex magnoliae officinalis), 24 components of Huangbai (cortex phellodendri), 3 components of Jianghuang (rhizoma curcumae longae), and 2 components of Tianhuafen (radix trichosanthis kirlowii). After combining them and removing the duplicates, 148 compounds were obtained, considered to be the active ingredients of RJP (See Table S1, http://links.lww.com/MD/I830, Supplemental Digital Content, which demonstrates drug ingredient target relationships).

### 3.2. Prediction of scattered target points of RJP

The TCMSP database was used to obtain the predicted targets of each drug component, and the weights were then removed to get 270 target protein names. After weight removal, 226 related gene names were obtained using the Uniport database for protein-gene name conversion. These genes were considered to represent the targets of the active ingredients of RJP in the human body.

### 3.3. Monkeypox target prediction

**Figure 2.**
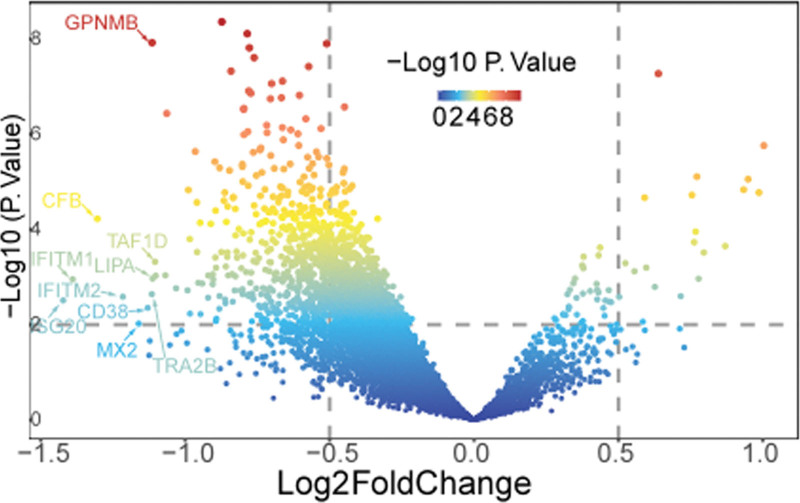
Volcano plot for differential analysis of GSE24125 dataset for monkeypox.

We obtained 560 eligible DEGs that met the threshold mentioned above, 540 of which were down-regulated while the remaining were up-regulated (See Table S2, http://links.lww.com/MD/I831, Supplemental Digital Content, which illustrates detailed data). The gradual volcano plot depicts the distribution of the top 10 DEGs (Fig. 2). The above 560 DEGs of the MPXV were used to intersect with the target gene of RJP to obtain the intersection targets.

### 3.4. RJP-monkeypox common targets analysis

**Figure 3.**
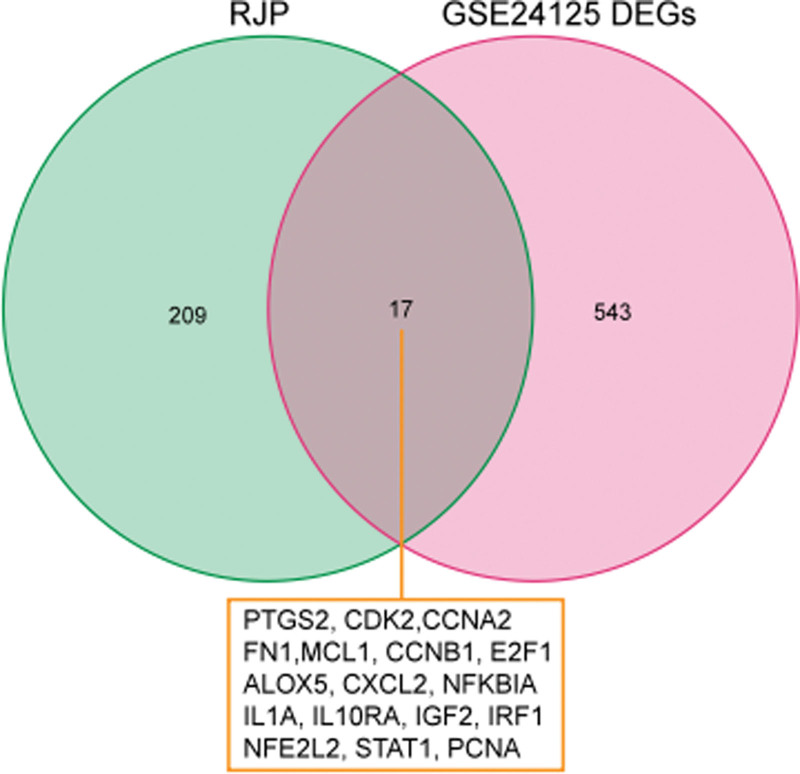
Venn diagram of the common targets of RJP and monkeypox. RJP = Ruyi Jinhuang Powder.

**Figure 4.**
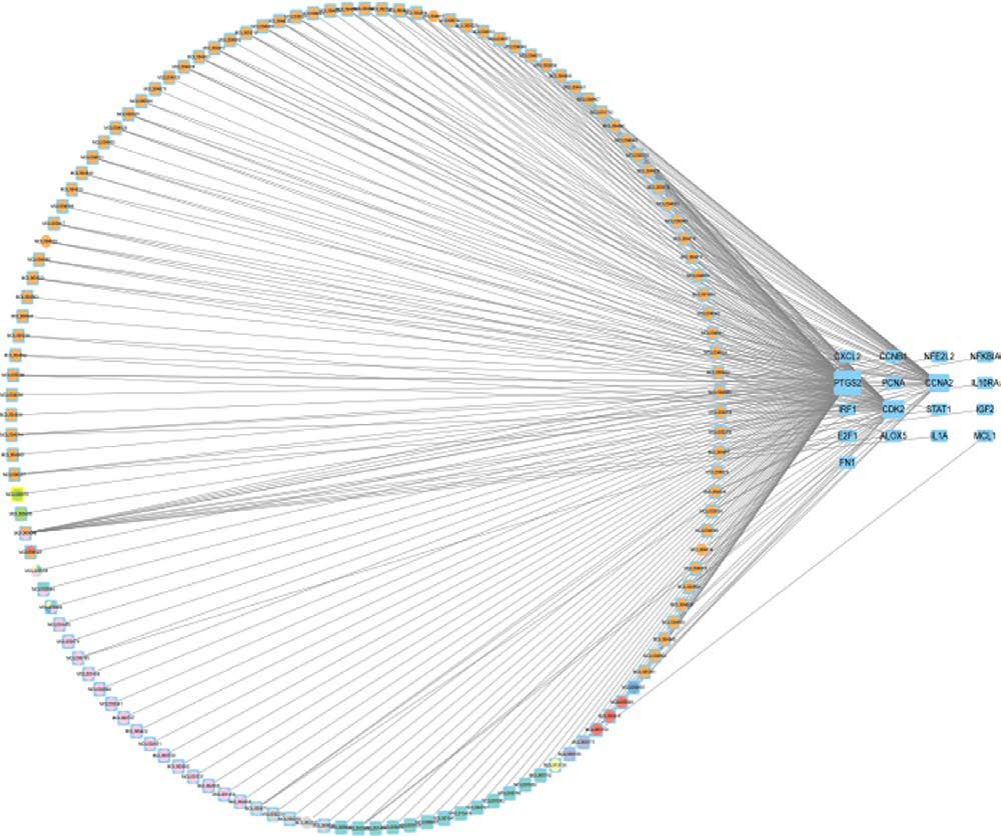
Drug composition target map. The number of colors in the circle represent the number of small molecule compounds corresponding to Chinese medicine. The right side of the diagram represent the drug gene target.

After the intersection operation, RJP and monkeypox had 17 common intersectional targets, which, in our opinion, were the effective sites of RJP on monkeypox (Fig. 3). The drug composition target map is displayed in Figure 4.

### 3.5. GO, KEGG, and DO analysis by bioinformatics

**Figure 5.**
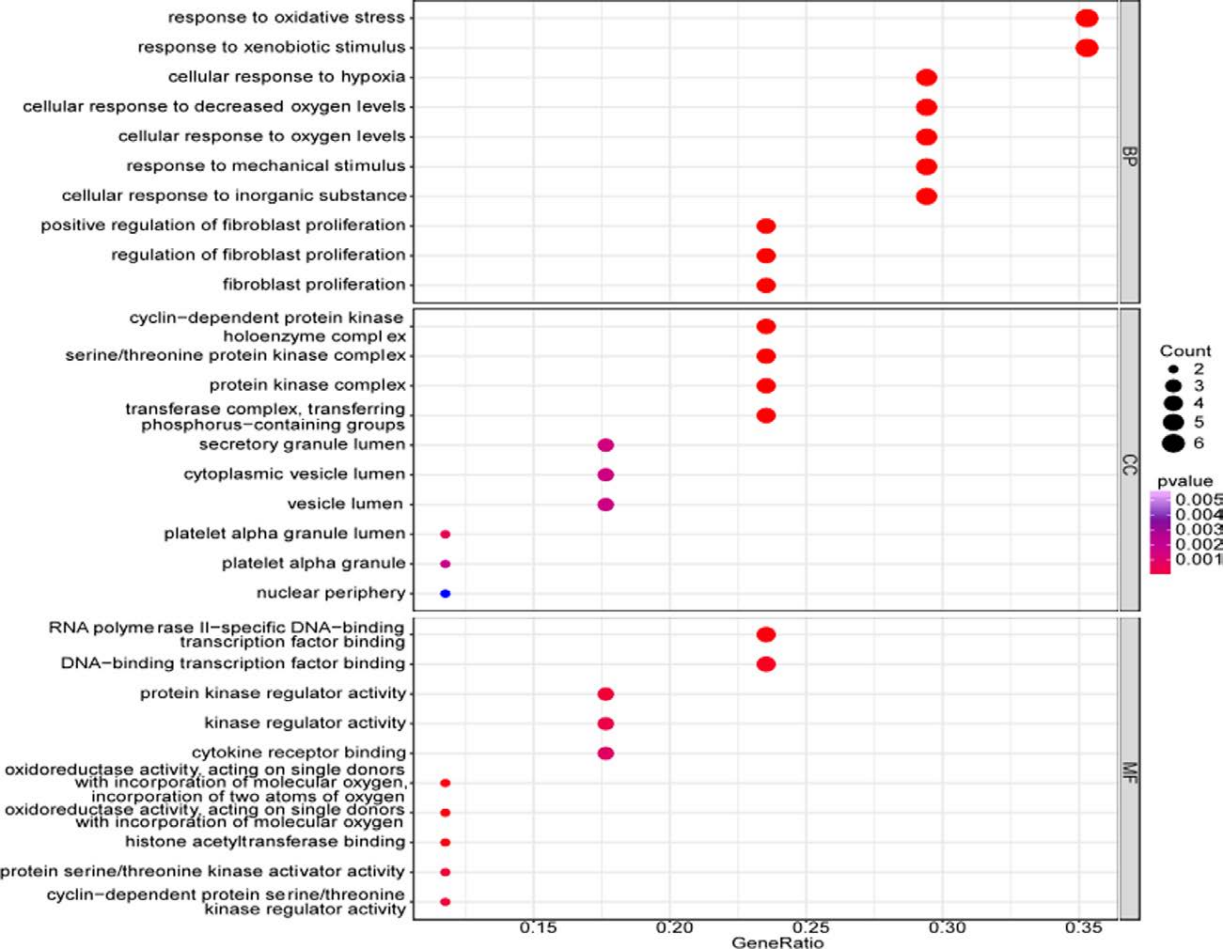
Bubble chart of the top 10 results of GO enrichment analysis. The bubble size represent the number of genes enriched and the bubble color represent the *P* value. GO = gene ontology.

As is common knowledge, GO analysis includes biological processes (BP), cellular components (CC), and molecular functions (MF). We obtained 754 entries (See Table S3, http://links.lww.com/MD/I832, Supplemental Digital Content, which illustrates detailed data about GO analysis), including 671 for BP, 23 for CC, and 60 for MF. The bubble graph in Figure 5 displays the top 10 significant enrichment items with the highest gene counts in BP, CC, and MF. The results demonstrated that RJP is mainly used to treat monkeypox by targeting positive regulation of fibroblast proliferation (GO: 0048146), cellular response to hypoxia (GO: 0071456), reactions of BP like a cellular response to decreased oxygen levels (GO: 0036294), cyclin-dependent protein kinase holoenzyme complex (GO:0000307), and serine/threonine-protein kinase complex (GO:1902554), CC such as protein kinase complex (GO: 1902911), RNA polymerase II-specific DNA-binding transcription factor binding (GO:0061629), oxidoreductase activity, acting on single donors with incorporation of molecular oxygen, incorporation of 2 atoms of oxygen (GO:0016702), oxidoreductase activity, acting on single donors with incorporation of molecular oxygen (GO: 0016701), etc.

**Figure 6.**
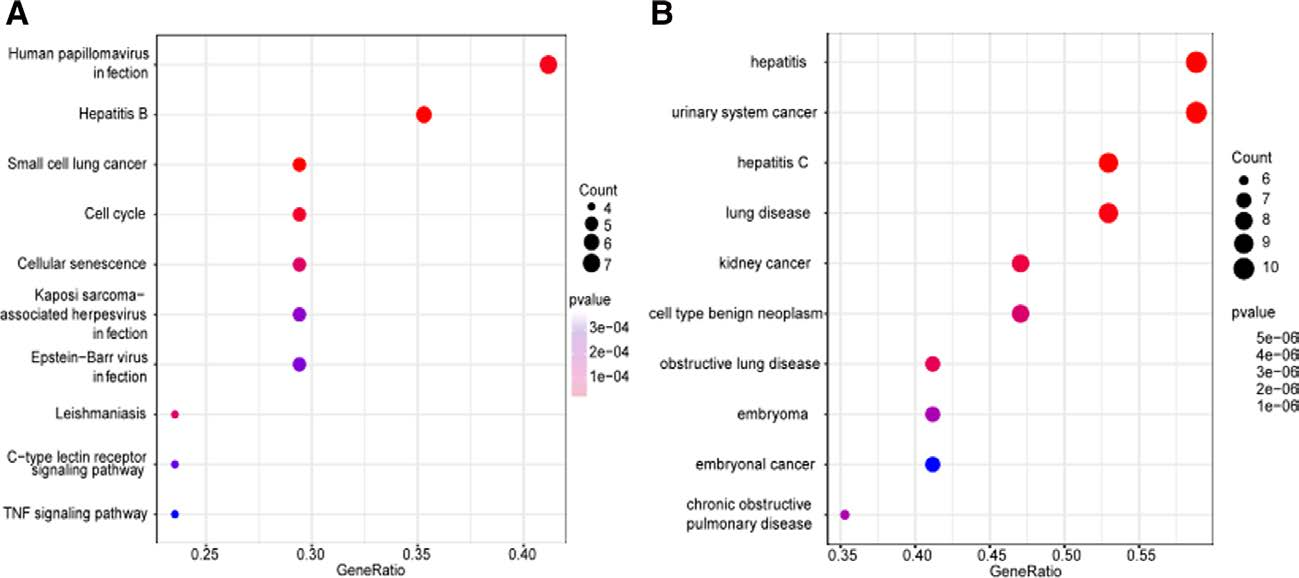
KEGG, DO enrichment analysis. (a) KEGG top ten pathways. (b) DO enrichment analysis of the top 10 pathways. DO = disease ontology, KEGG = Kyoto Encyclopedia of Genes and Genomes.

The function and signal transduction pathway of the anti-target of RJP were studied using the KEGG pathway. In this study, we acquired 60 KEGG pathways (See Table S4, http://links.lww.com/MD/I833, Supplemental Digital Content, which illustrates detailed data about KEGG pathway analysis). The first 3 statistically significant pathways were cellular senescence (hsa04218), hepatitis B (hsa05161), and leishmaniasis (hsa05140). The top 10 significant enrichment items in the pathway with the highest gene counts are displayed in the bubble plot in Figure 6A.

Common targets and major diseases were identified using DO enrichment analysis, and 227 statistically significant related diseases were determined (See Table S5, http://links.lww.com/MD/I834, Supplemental Digital Content, which illustrates 227 diseases). The top 10 DO results are exhibited in Figure 6B. Among them, the top 3 are hepatitis C (DOID: 1883), hepatitis (DOID: 2237), and urinary system cancer (DOID: 3996).

### 3.6. PPI network

**Figure 7.**
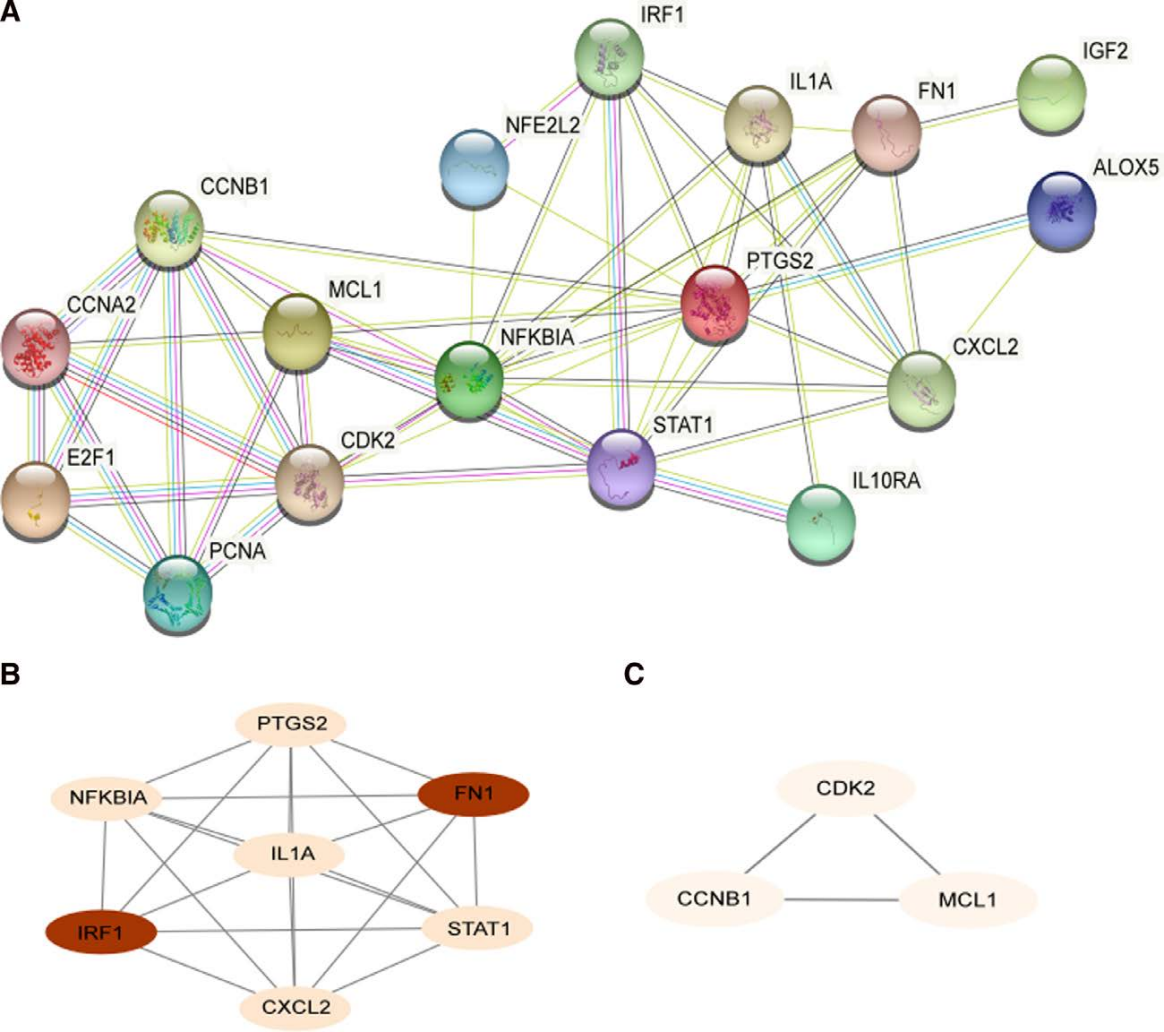
PPI network diagram and MCODE module diagram. (a) PPI network diagram. The network contained 17 targets. (b) MCODE Module Cluster 1 with a score of 6.667. (c) MCODE Module Cluster 2 with a score of 3. PPI = protein-protein interactions.

**Figure 8.**
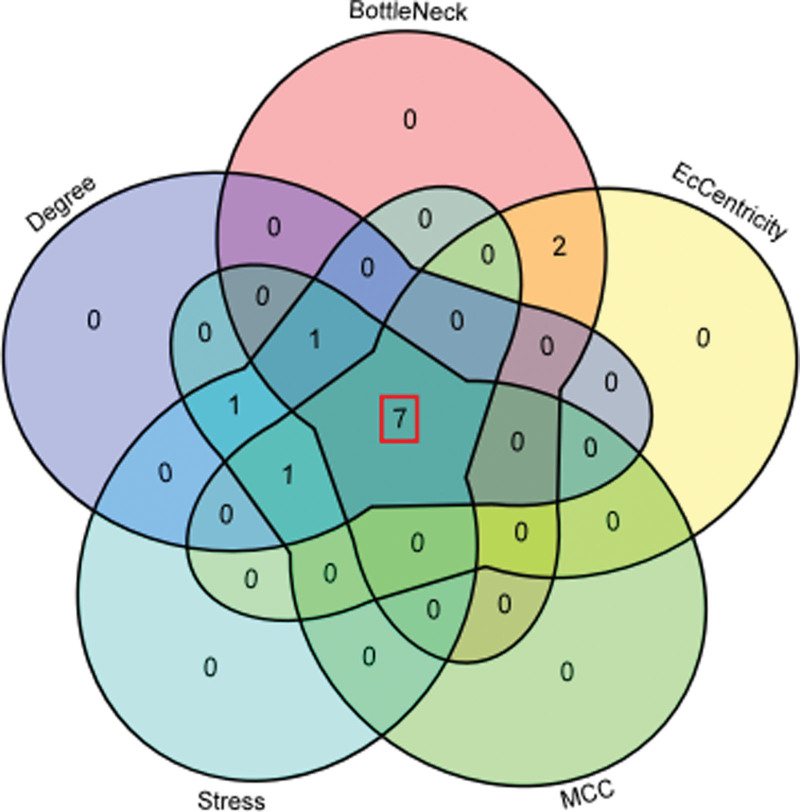
Venn diagram of monkeypox-RJP core target screening. RJP = Ruyi Jinhuang Powder.

After uploading the data to the STRING database, a protein interaction network with 17 points and 51 edges was obtained (Fig. 7A). Two functional clustering modules were obtained using the MCODE plug-in, with module 1 scoring 6.667 points and module 2 scoring 3 points (Fig. 7B and C). After further analysis using Degree, BottleNeck, EcCentricity, maximal clique centrality, and Stress in the Cytohubba plug-in, 7 core targets were obtained: cyclin-dependent kinase 2 (CDK2), fibronectin 1 (FN1), signal transducer and activator of transcription 1 (STAT1), prostaglandin-endoperoxide synthase 2 (PTGS2), interferon regulatory factor 1 (IRF1), interleukin 1-alpha (IL1A), and C-X-C motif ligand 2 (CXCL2) (Fig. 8 and Table 1).

**Table 1:** Cytohubba 5 algorithm scores: Degree, BottleNeck, EcCentricity, MCC, and stress.

Degree	Score	BottleNeck	Score	EcCentricity	Score	MCC	Score	Stress	Score
PTGS2	11	PTGS2	11	FN1	0.5	PTGS2	320	PTGS2	160
NFKBIA	10	STAT1	3	STAT1	0.5	NFKBIA	318	CDK2	134
STAT1	9	MCL1	3	PTGS2	0.5	STAT1	290	NFKBIA	120
CDK2	9	FN1	2	CDK2	0.5	IL1A	242	STAT1	94
FN1	7	CXCL2	2	NFKBIA	0.5	CXCL2	242	FN1	70
IL1A	7	CDK2	2	ALOX5	0.3333333	FN1	145	CCNB1	68
CXCL2	7	ALOX5	1	IRF1	0.3333333	IRF1	126	MCL1	68
CCNB1	7	IRF1	1	IL1A	0.3333333	CDK2	120	IL1A	30
MCL1	7	IL1A	1	CXCL2	0.3333333	CCNB1	72	CXCL2	20
IRF1	6	CCNA2	1	CCNA2	0.3333333	MCL1	72	IRF1	10

CDK2 = cyclin-dependent kinase 2, CXCL2 = C-X-C motif ligand 2, FN1 = fibronectin 1, IL1A = interleukin 1-alpha, IRF1 = interferon regulatory factor 1, MCC = maximal clique centrality, PTGS2 = prostaglandin-endoperoxide synthase 2, STAT1 = signal transducer and activator of transcription 1.

### 3.7. Molecular docking results

**Figure 9.**
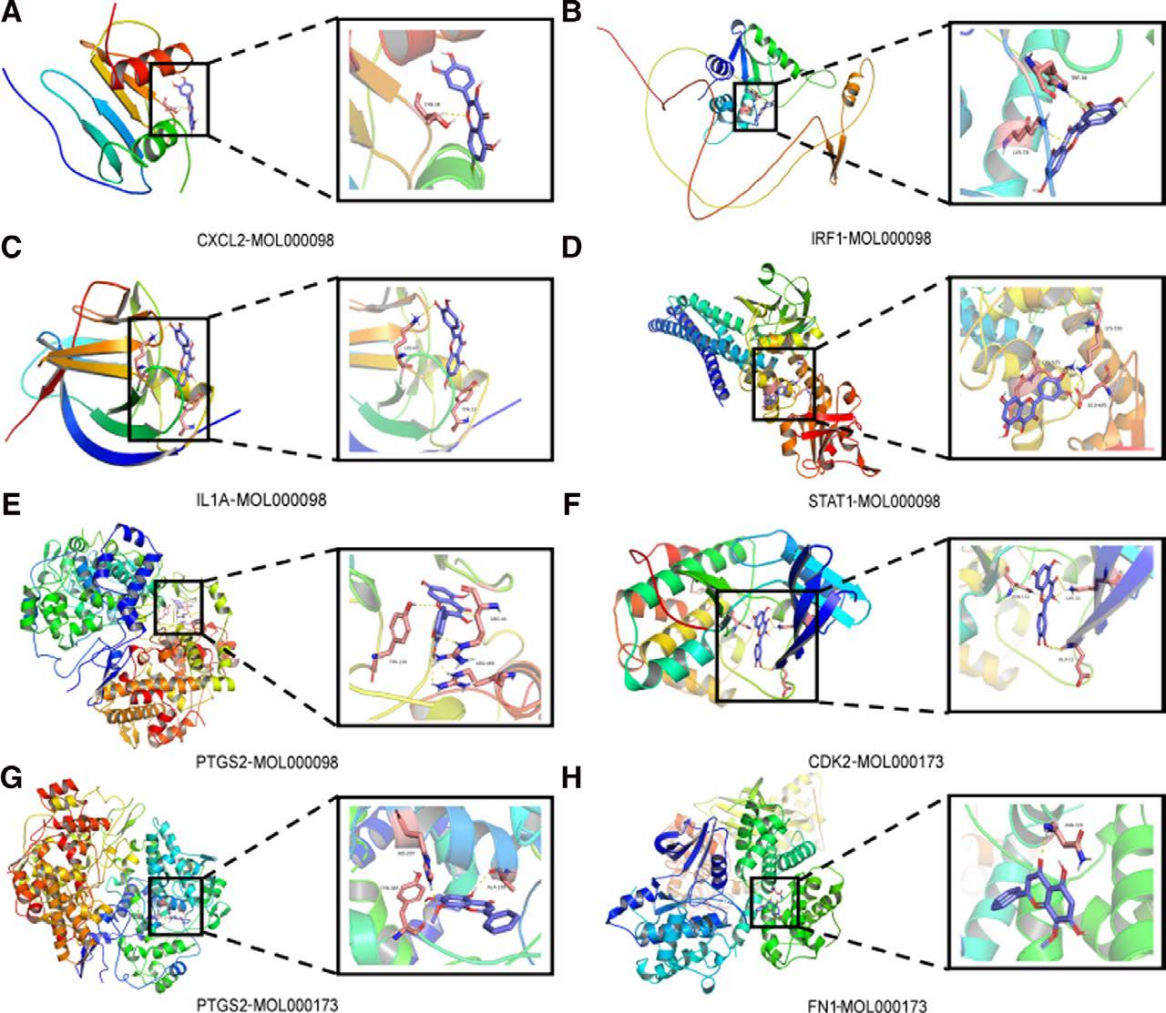
Molecular docking. (A) CXCL2-MOL000098. (B) IRF1-MOL00098. (C) IL1A-MOL000098. (D) STAT1-MOL00098. (E) PTGS2-MOL000098. (F) CDK2-MOL000173. (G) PTGS2-MOL000173. (H) FN1-MOL000173. CDK2 = cyclin-dependent kinase 2, CXCL2 = C-X-C motif ligand 2, FN1 = fibronectin 1, IL1A = interleukin 1-alpha, IRF1 = interferon regulatory factor 1, PTGS2 = prostaglandin-endoperoxide synthase 2, STAT1 = signal transducer and activator of transcription 1.

Subsequently, 2 candidate bioactive compounds (wogonin, quercetin) were docked with 7 candidate target proteins, including STAT1 (PDB ID: 7nuf), FN1 (PDB ID: 6gue), IL1A (PDB IDs: 2kki), CXCL2 (PDB IDs: 1qnk), CDK2 (PDB ID: 1b38), PTGS2 (PDB ID: 5f19), and IRF1 (UniProt ID: AF-P10914-F1-model _ v2). The results are displayed in Figure 9. Meanwhile, the binding model between the target proteins mentioned above and small molecule compounds is illustrated (See Fig. S1, http://links.lww.com/MD/I835, Supplemental Digital Content, which illustrates the binding model between the target proteins and small molecule compounds).

Binding energies are often calculated to assess the degree of affinity of a component for a protein target. Generally, binding energies of less than −4.25, −5.0, or −7.0 kcal/mole represent fair, good, or strong binding activity between the ligand and receptor, respectively. The binding energy indicates the possibility that the receptor will bind to the ligand. Conversely, lower binding energy implies a higher ligand-receptor affinity and a more stable conformation. According to the results of binding energy docking in Table 2, these components bound well to the active site of the protein target. The results demonstrated that PTGS2 had the lowest binding energy (−9.4 kcal/mol), while STAT1 had the highest binding energy (−4.9 kcal/mol), and the average binding energy was −7.35 kcal/mol. It demonstrated that the 2 representative compounds of RJP could effectively bind to the 7 primary monkeypox targets, which may play a key role in treating monkeypox infection.

**Table 2:** Molecular docking ligand-receptor comparison table.

MOL ID	Target	RJP	Receptor	Binding energy (kcal/mol)
MOL000098	STAT1	Huangbai, Gancao	7nuf	−4.9
MOL000098	PTGS2	Huangbai, Gancao	5f19	−9.4
MOL000098	IRF1	Huangbai, Gancao	AF-P10914-F1-model_v2	−6.8
MOL000098	IL1A	Huangbai, Gancao	2kki	−6.6
MOL000098	CXCL2	Huangbai, Gancao	1qnk	−5.8
MOL000173	CDK2	Cangzhu	1b38	−8.6
MOL000173	FN1	Cangzhu	6gue	−7.6
MOL000173	PTGS2	Cangzhu	5f19	−9.1

CDK2 = cyclin-dependent kinase 2, CXCL2 = C-X-C motif ligand 2, FN1 = fibronectin 1, IL1A = interleukin 1-alpha, IRF1 = interferon regulatory factor 1, PTGS2 = prostaglandin-endoperoxide synthase 2, RJP = Ruyi Jinhuang Power, STAT1 = signal transducer and activator of transcription 1.

## 4. Discussion

Activities aimed at reducing high mortality rates during the COVID-19 pandemic were emphasized. Smallpox is caused by a smallpox virus infection, which has high transmission and mortality rates. With the focus on vaccination, smallpox has been eliminated. As a result, modern humans have little immunity to smallpox. Monkeypox is an infectious disease that has gradually spread worldwide recently. It is closely related to the smallpox virus and can cause similar human clinical diseases.

The finding that treatment with antiviral drugs beginning 24 hours after monkeypox infection significantly reduces the death rate and the number of monkeypox-related lesions;^[32]^ supports the potential of antiviral therapy for monkeypox. Recent studies have shown that Tecovirimat and Brincidofovir exhibit antiviral activity against double-stranded DNA viruses, including poxviruses.^[33]^ However, due to the mutability of viruses, specific medicine against viruses often develop drug resistance after prolonged use. Therefore, finding suitable medication to prevent and treat monkeypox in the future is crucial. In previous studies, we have found that antiviral treatment for MPXV may be better than vaccination.^[32]^

Due to the multi-target effect and relatively safe characteristics, TCM has attracted growing interest in recent years. RJP is a regularly prescribed drug in TCM. It has a variety of drug components and is often used for treating herpes and fever in conventional treatments, with the advantages of multi-target synergistic therapy and low toxicity. In this study, the potential therapeutic targets of RJP for monkeypox were explored through network pharmacology and multiple databases, and the associated pharmacological mechanisms were predicted. As we previously mentioned, TCM has demonstrated great potential for viral suppression, which can be therapeutic in various viral infectious diseases, including HIV, COVID-19, and others.^[34,35]^ Recently, network pharmacology research has provided an effective strategy for obscuring the molecular mechanism of multi-target drugs. We used target prediction, GO, KEGG, DO, PPI, and other analysis methods to explore the pathways, components, and targets of RJP in the treatment of monkeypox. 7 core targets were screened after merging the PPI network of related RJP and monkeypox targets. GO and KEGG enrichment analysis confirmed that RJP could regulate various immunological and antiviral-related signals to conduct antiviral therapy, including TNF, age range, c-type lectin receptor signaling pathways, Kaposi sarcoma-associated herpesvirus infection, etc. Modulating these signaling pathways was a promising area of research and it provided a new direction for treating monkeypox.

A total of 148 active components and 17 hub targets were identified in this research. In previous studies about these active ingredients, wogonin has been revealed to reduce inflammation by inhibiting the production of inflammatory factors by immune cells such as lymphocytes, macrophages, and neutrophils.^[32]^ Additionally, some studies have revealed that it can inhibit herpes virus infection by regulating the cellular NF-KB and MARK pathways.^[36]^ Quercetin is a kind of polyphenolic flavonoid, and subsequent studies showed it has anti-viral activity against the influenza virus, hepatitis C virus, dengue virus, and other different viruses.^[37–40]^ However, quercetin has historically had difficulty becoming a more practical clinical medicinal agent due to its poor solubility. According to some researchers, it can enhance the antiviral activity of cells after being specially prepared.^[41]^ Mechanism studies showed that quercetin was selected as an inhibitor against SARS-CoV-2 which was mediated by blocking the 3a channel, inhibiting protein kinase B and phosphorylating protein kinase.^[42,43]^ Other studies showed that the compound also has the function of regulating immune responses by reducing pro-inflammatory cytokines and enhancing anti-inflammatory cytokines. These have been proven to affect COVID-19 treatment positively.^[44–46]^ Wu et al found quercetin inhibited influenza infection by interacting with the HA2 subunit.^[38]^ According to previous reports, RJP can treat diabetic repair ulcers through the Wnt/β-catenin pathway and can play an anti-inflammatory role by down-regulating IL1A, which is consistent with the key nodes derived from our PPI protein interaction.^[47]^ Moreover, RJP wound healing is the reaction of cells to injury; fibronectin is one of the key proteins in dynamic wound healing. MPXV replicates at the initial infection site, which causes an inflammatory response.^[48]^ More specifically, the virus presence significantly affects T-cell-mediated cytokine responses.^[49]^ The bioactive compounds found in RJP might suppress monkeypox by directly inhibiting the virus and lowering inflammation caused by monkeypox infection.

Our study identified 7 potential therapeutic targets for monkeypox, including CDK2, FN1, STAT1, PTGS2, IRF1, IL1A, CXCL2. However, there has been insufficient research on therapeutic targets and biomarkers for monkeypox. Therefore, there has been no relevant experiment to prove the role of these 7 genes in monkeypox. Encouragingly, the molecular docking results showed that most of the hub targets and the corresponding ligands had a strong binding affinity, except that the docking scores of quercetin and STAT1 was more than −5 kcal/mol (−4.9 kcal/mol). On one hand, the results of molecular docking improve the accuracy of this study on the treatment of monkeypox with RJP. On the other hand, relevant literature discussions provide insights into the mechanistic studies of monkeypox.

FN1 is a member of the FN protein family. The FN-containing EDA significantly affects skin wound healing.^[50]^ Catalase and other substances help remove excess superoxide dismutase and catalase in the body and slow aging. A significant role of FN1 in anti-inflammatory mechanisms and related diseases has been extensively reported in the literature.^[51–53]^ FN1 is highly expressed in nodular disease, a systemic inflammatory disease manifested by pulmonary involvement and erythema nodosum of the skin.^[33]^

CDK2 is involved in cell cycle regulation and is generally considered important in cancer pathogenesis.^[54]^ However, CDK2 also has prominent contributions to immunological diseases and anti-inflammatory mechanisms. Evidence suggests that CDK2 is involved in the expression of inflammatory mediators in the psoriatic epidermis and regulates the psoriatic epidermal cell cycle,^[55]^ suggesting that modulating CDK2 is an effective strategy for treating psoriasis.^[56]^ This information undoubtedly provides us insight into studying the control of monkeypox.

STAT1 have been demonstrated to be involved in regulating cellular antiviral defense mediated by linear ubiquitination.^[57]^ It can reduce the structural damage caused by influenza virus infection through the JAK1/2-STAT1 signaling pathway. This pathway induces inflammatory response and regulates immunological response by mediating the signal transduction of the IL-2, interleukin-6, IL-10, interferon families, as well as other cytokines and growth factors.^[58]^

IRF1 is a transcription factor that can be produced by the IFN-γ activated JAK/STAT1 signaling pathway. IRF1 expression is significantly elevated in IFN-γ-activated macrophages, which regulate the expression of IL-12 and Nitric Oxide. Zafar et al found that the STAT1-IRF1 signaling process regulated the expression of pro-inflammatory genes in atherosclerosis, a pathogenic factor in the disease, and that targeted knockdown of IRF1 expression was beneficial in limiting plaque development.^[59]^

PTGS2 gene encodes the COX-2 protein in organisms associated with inflammation.^[60]^ COX-2 is one of the important rate-limiting enzymes that catalyze the production of prostaglandins from arachidonic acid. The expression of COX-2 is rapidly up-regulated when cells are stimulated by various factors, including growth factors, cytokines, inflammatory mediators, etc. PGE2 is the main product of COX-2-catalyzed arachidonic acid production.

CXCL2 is an inflammatory chemokine, and its receptor CXCR2 is mainly expressed on the surface of neutrophils and natural killer cells when CXCL2 binds to CXCR2, its expression activity increases, which induces inflammatory cells such as neutrophils, monocytes, lymphocytes, and natural killer cells to migrate to the injury site and accumulate in the inflammatory site, aggravating the inflammatory response at the injury site.^[61]^

A potent mediator of inflammation and immunological response, IL1A belongs to the interleukin 1 family and is synthesized by various cell types, including macrophages.^[62]^ Evidence suggests that IL1A plays an important role in inflammatory diseases such as cardiovascular disease, colonic inflammation, neuroinflammation, and human skin inflammation.^[63]^ Lukens et al found a critical role for IL1A in tissue damage and wound inflammation in the severe inflammatory syndrome of neutrophilic skin disease.^[50]^

Some of these proteins regulate the damage caused by the virus from the perspective of inflammation and immunity to reduce viral replication, and some others directly played an antiviral role with a variety of targets and ways to reduce the damage by MPXV on the human body. However, there are some limitations to our study. First, limited by the source problem of monkeypox samples, the MPXV genes in this study were derived from secondary mining of the GEO database. Second, we could not experimentally verify the effectiveness of RJP against the MPXV due to the specificity of the disease and the safety concern surrounding virus prevention and control. This study can provide some significance for future research on monkeypox control and mechanism as scientists continue to investigate monkeypox in greater detail.

## 5. Conclusions

In conclusion, this study found that RJP has great potential in treating monkeypox using a network pharmacological approach and bioinformatic analysis. It has the characteristics of multi-component, multi-target, multi-pathway, and complex connection. Enrichment analysis revealed that RJP has various synergistic therapeutic effects on monkeypox. The specific mechanism of RJP in the treatment of monkeypox may play a positive role through anti-inflammatory and antiviral activities. The limitation of this study is that there is no experimental verification of the interaction, compatibility changes, and different contents of the components in the compound on monkeypox. Afterward important pharmaceutical components should be selected for further mechanism analysis. Nevertheless, it can provide ideas for future research and experimental verification of the corresponding molecular mechanism. It also provided great clinical significance for the follow-up search to find more clinical drugs for treating monkeypox and other viral infectious diseases.

## Author contributions

**Conceptualization:** Xi Zhang.

Data curation: Xinping Yu.

Methodology: Chengcheng Fan.

Project administration: Shuo Zhang.

Resources: Huan Li.

Software: Yingkai Shen.

Supervision: Shuo Zhang.

Validation: Zijin Sun.

Visualization: Yueming Li.

Writing – original draft: Xi Zhang.

Writing – review & editing: Zhichao Yu.

## Supplementary Material












