# Supplementary figures and images for: Network pharmacology and bioinformatics to identify molecular mechanisms and therapeutic targets of Ruyi Jinhuang Powder in the treatment of monkeypox
Source: Medicine (Baltimore). 2023 Apr 28;102(17):e33576. doi: 10.1097/MD.0000000000033576 (PMC10145999; doi:10.1097/MD.0000000000033576)

**Figure S1.** The binding model between the target proteins and small molecule compounds.

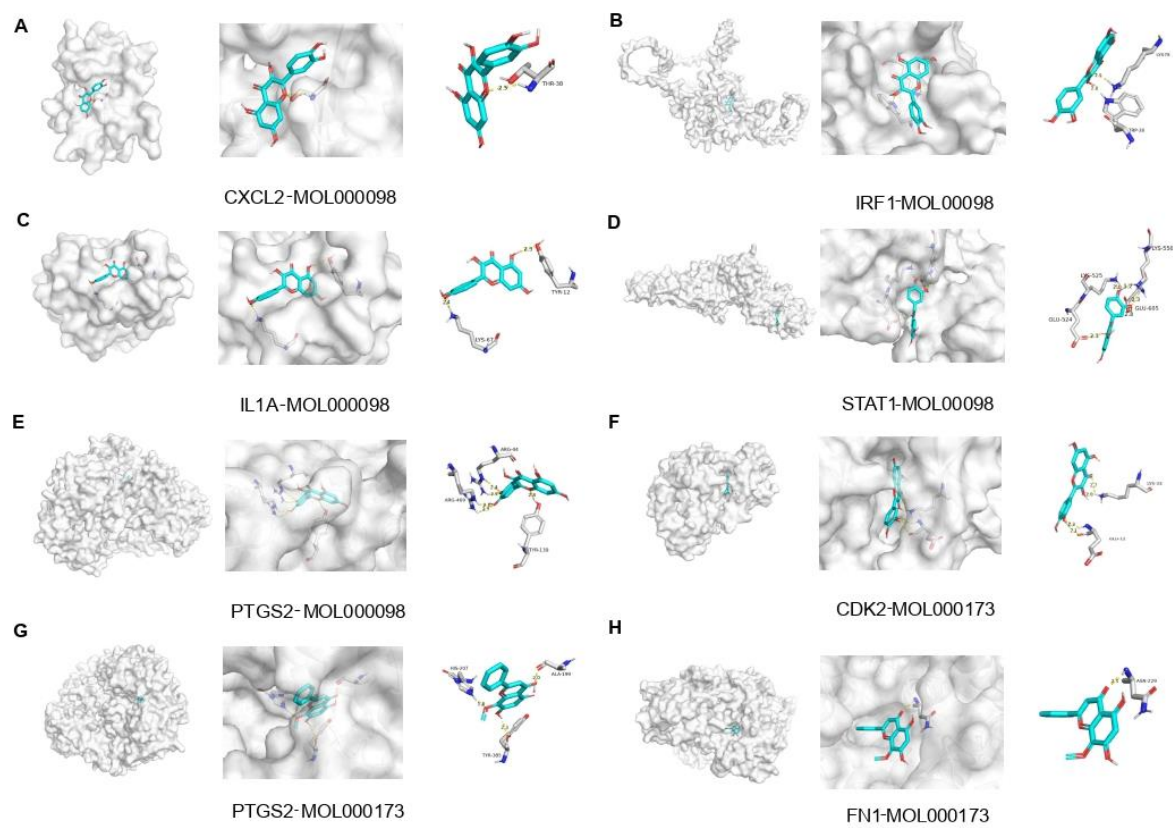

Supplement: Supplementary file 6 [file medi-102-e33576-s006.pdf]
